# Nomogram-Based Prediction Model for Postherpetic Neuralgia in Immunosuppressive Patients

**DOI:** 10.3390/jcm15093435

**Published:** 2026-04-30

**Authors:** Xiao-Yuan Pan, Li-Na Lu, Jing Wang, Li-Hong Mei, Gao Yang

**Affiliations:** 1 Department of Dermatology, Jinshan Hospital of Fudan University, Shanghai 201508, China; panxiaoyuan1999@163.com (X.-Y.P.); l35820018@163.com (L.-N.L.);; 2 Department of Dermatology, Zhongda Hospital of Southeast University, Nanjing 210009, China

**Keywords:** immunosuppression, herpes zoster, postherpetic neuralgia, inflammatory marker, risk factors

## Abstract

**Background/Objectives**: Herpes zoster is caused by varicella-zoster virus reactivation, which often leads to a chronic pain condition named postherpetic neuralgia (PHN). Patients with immunosuppressive conditions face a heightened risk of developing PHN. This study aims to identify factors contributing to PHN development in immunosuppressive patients. **Methods**: This retrospective cohort study was conducted involving 219 immunosuppressive patients from two centers and split into training and test cohorts. Participants were divided into PHN (*n* = 88) and acute phase pain (ACP, *n* = 131) groups. Univariate and multivariate logistic regression analyses were used to identify clinical predictors of PHN. A nomogram was constructed to predict PHN risk by integrating significant predictors. The discrimination, calibration and clinical usefulness of the nomogram were evaluated. **Results**: Multivariate analysis revealed metabolic syndrome, older age, higher lactate dehydrogenase (LDH), and higher neutrophil-to-lymphocyte ratio (NLR) as significant PHN predictors. The nomogram showed good discrimination in both training (AUC of 0.83 [95% CI 0.77–0.90] with a specificity of 0.78, sensitivity of 0.87, NPV of 0.90, and PPV of 0.73) and test cohorts (AUC of 0.85 [95% CI 0.75–0.96] with a specificity of 0.82, sensitivity of 0.85, NPV of 0.89, and PPV of 0.76). Clinical decision curve analysis confirmed the practical utility of the nomogram. **Conclusions**: The nomogram incorporating age, metabolic syndrome, LDH, and NLR are useful in estimating PHN risk among immunosuppressed patients.

## 1. Introduction

Herpes zoster is caused by the reactivation of the varicella-zoster virus in the dorsal root or cranial nerve ganglia, which manifests as painful papules and vesicles along nerve distributions [1]. Postherpetic neuralgia (PHN) is the most common complication of herpes zoster. Defined by the International Association for the Study of Pain, PHN refers to the chronic pain persisting for over three months after the acute phase [2]. PHN frequently presents as burning, stabbing, or electric shock-like pain with hyperalgesia or allodynia. Patients with PHN often suffer from functional impairment, sleep disturbances, emotional disorders like anxiety and depression, which significantly reduce quality of life [3].

Epidemiological surveys indicate that the annual incidence of herpes zoster is 3–5 per 1000 individuals. The risk of patients with herpes zoster progressing to PHN ranges from 5% to 30% [4]. Existing research reports that inflammation is generally involved in PHN patients with herpes simplex, diabetes, and immunosuppressive conditions [5]. While immunosuppressive conditions can reduce immune responses, inflammation can still occur, particularly through mechanisms involving neutrophils [6]. Previous studies identified an increased risk of PHN in patients with immunosuppressive conditions [7]. However, few studies examine the risk factors influencing PHN development in patients with immunosuppressive conditions.

Inflammatory markers are simple, and emerging complete blood count-derived markers reflecting systemic inflammation, which mainly includes neutrophil-to-lymphocyte ratio (NLR) and platelet-to-lymphocyte ratio (PLR) [8]. These inflammatory markers have been shown in previous studies to more accurately reflect the body’s inflammatory and immune conditions compared to traditional single clinical indicators. These markers have significant value in predicting the severity and prognosis of inflammation-related diseases [9].

Although these inflammatory ratios have been widely applied as systemic inflammation markers, their predictive role in PHN among immunosuppressed populations has not been systematically examined. To address this gap, our study aims to investigate the factors contributing to the development of PHN in patients under immunosuppressive conditions, with the goal of providing better prevention strategies for clinical practice.

## 2. Materials and Methods

### 2.1. Ethics Considerations

This retrospective study was conducted in accordance with the Declaration of Helsinki and approved by the Ethics Committee of Jinshan Hospital of Fudan University (JIEC2025-S80) and Zhongda Hospital (2023ZDSYLL394-P01). Written informed consent was waived by the Ethics Committee due to the retrospective nature of this study.

### 2.2. Patients and Study Design

From January 2020 to October 2024, consecutive patients diagnosed with immunosuppressive conditions and presenting with herpes zoster in the Dermatology Department of two centers (Center A and Center B) were retrospectively analyzed. The data from Center A and Center B were used as a training and test cohort, respectively. The patients were divided into a PHN group and an acute phase pain (ACP) group.

The inclusion criteria were as follows: meeting the definition of immunosuppressive conditions; herpes zoster infection after the diagnosis of the immunosuppressive condition. The exclusion criteria were as follows: lost to follow-up; incomplete medical history and laboratory tests. All patients included in the analysis completed a minimum 3-month follow-up period. PHN was defined as pain persisting ≥ 3 months after rash onset with the Visual Analogue Scale (VAS) score > 0.

Immunosuppressive conditions were defined as alterations in cellular and/or humoral immunity due to the patient’s underlying disease or treatment. This included patients treated (receipt of any systemic antitumor therapy including chemotherapy, radiotherapy, immunotherapy, targeted therapy, or systemic corticosteroids within 6 months before herpes zoster onset) or untreated (confirmed diagnosis of malignancy with no antitumor therapy initiated before herpes zoster onset) malignant tumors.

### 2.3. Clinical Data Collection

Data were collected through the hospital’s electronic medical record system. General clinical information was collected, including gender, age, relationship between pain and rash, nerve involvement, lesion distribution type, metabolic syndrome (hypertension, diabetes mellitus or hyperlipidemia), time of blister cessation, scab formation time, time from onset to admission, frequency of painkillers, and time of hospital stay.

### 2.4. Laboratory Tests

Laboratory test results were collected, including hemoglobin, platelet count, lactate dehydrogenase (LDH), erythrocyte sedimentation rate, red cell distribution width, fibrinogen, albumin, prealbumin, neutrophil count, lymphocyte count, C-reactive protein, high-density lipoprotein, low-density lipoprotein, triglycerides, and cholesterol.

The inflammatory marker values were calculated as follows: NLR (neutrophil-to-lymphocyte ratio), neutrophil count divided by lymphocyte count; PLR (platelet-to-lymphocyte ratio), platelet count divided by lymphocyte count; PNR (platelet-to-neutrophil ratio), platelet count divided by neutrophil count; RDW/Hb, red cell distribution width divided by hemoglobin; CAR (C-reactive protein to albumin ratio), C-reactive protein divided by albumin and AFR (albumin-to-fibrinogen ratio), albumin divided by fibrinogen.

### 2.5. Clinical Predictor Selection and Nomogram Building

Using the training cohort data, univariate logistic regression analyses were first performed to identify variables significantly associated with PHN (*p* < 0.10). Variables meeting this threshold were subsequently entered into a multivariate logistic regression model to determine independent predictors. Before multivariate modeling, all candidate predictors were assessed for multicollinearity using the variance inflation factor (VIF); variables with VIF > 5 were excluded to ensure model stability. Stepwise selection based on the Akaike Information Criterion was then applied to identify the most parsimonious model with optimal goodness of fit. To improve interpretability, continuous predictors were rescaled to represent clinically meaningful unit changes. The final independent predictors with stable estimates were used to construct the nomogram. Each variable in the nomogram was assigned a weighted point score proportional to its regression coefficient for individualized prediction of PHN risk.

### 2.6. Nomogram Discrimination and Calibration

The predictive ability of the nomogram in predicting PHN was evaluated using the area under the receiver operating characteristic (ROC) curve (AUC) in both the training and test cohorts. Calibration curve was used to assess the goodness of fit of the nomogram in the training and test cohorts.

### 2.7. Clinical Usefulness

A decision curve analysis (DCA) was conducted to evaluate the clinical practicality of the nomogram and to measure the net benefits it offers at various threshold probabilities. The clinical net benefit of the nomogram across threshold probabilities ranged from 0 to 1. Net benefit was calculated by weighting true positives against false positives at each threshold. The nomogram was compared against the ‘treat-all’ and ‘treat-none’ default strategies. DCA was applied to both the training and test cohorts.

### 2.8. Statistical Analysis

All statistical analyses were conducted using R software (Version 4.5.0). Continuous variables were reported as the mean with the corresponding standard deviation. Normality was assessed. Depending on the data distribution, continuous variable comparisons between groups were carried out using either Student’s *t*-test for normally distributed data or the Mann–Whitney U test for non-normally distributed data. For categorical variables, comparisons were performed using Chi-square tests. Correlation analyses were carried out using Spearman’s correlation coefficients. Statistical significance was defined as a two-tailed *p*-value of less than 0.05. The nomogram and calibration curves were constructed using the rms package. ROC curve analysis and AUC calculation were performed using the pROC package (Version 1.19). Decision curve analysis was conducted using the rmda package (Version 1.6) with bootstrap of 1000 repetitions.

## 3. Results

### 3.1. Patient Characteristics

A total of 219 patients were included, divided into a training cohort (ACP group: *n* = 92, PHN group: *n* = 62) and a test cohort (ACP group: *n* = 39, PHN group: *n* = 26).

In the training cohort, the ACP group had 57 females (62.0%) and 35 males (38.0%), while the PHN group had 38 females (61.3%) and 24 males (38.7%). The pain durations of herpes zoster were 7.5 ± 2.6 weeks for ACP group and 19.4 ± 6.7 weeks for PHN group. In the test cohort, the ACP group had 31 females (79.5%) and 8 males (20.5%), and the PHN group had 17 females (65.4%) and 9 males (34.6%). The pain durations of herpes zoster were 7.1 ± 2.3 weeks for ACP group and 20.0 ± 6.7 weeks for PHN group. No significant differences in gender distribution were observed between the ACP and PHN groups in either cohort (*p* > 0.05 for all comparisons).

**Figure 1:**
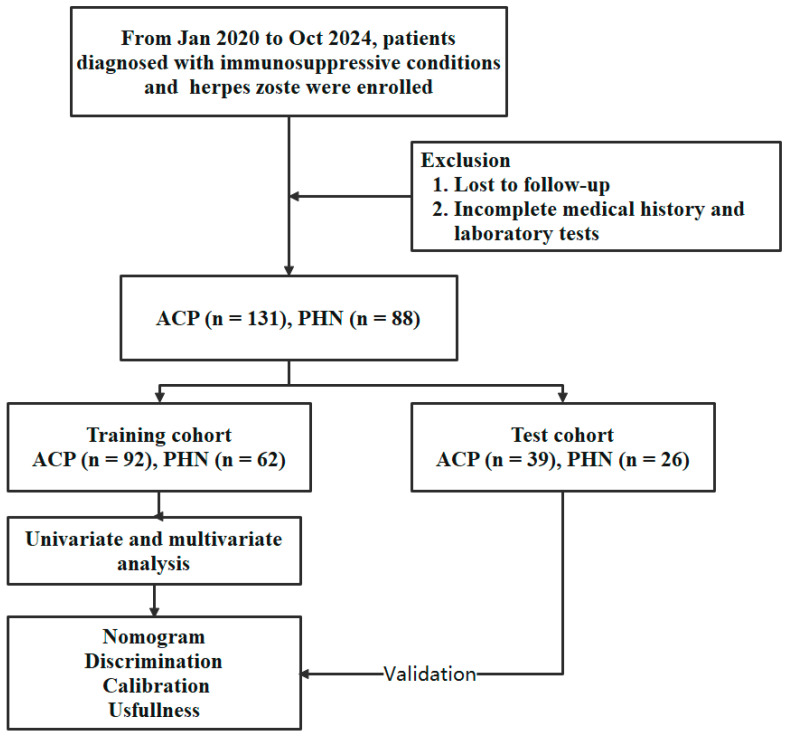
Study flowchart showing patient enrollment, allocation, and analysis workflow.

**Figure 2:**
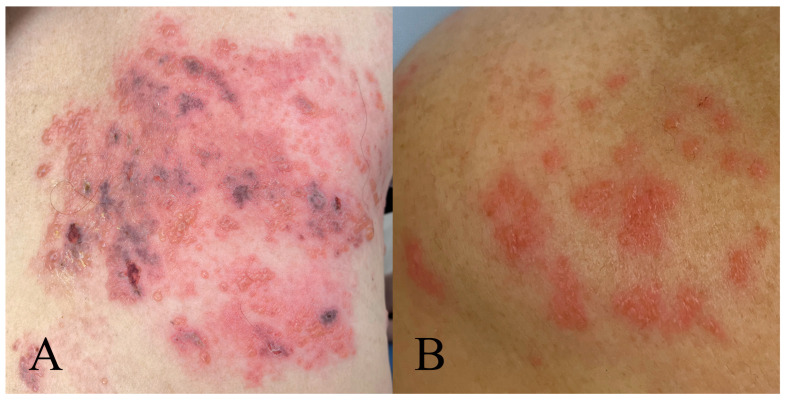
Clinical presentation of herpes zoster in immunosuppressive patients. Herpes zoster lesions in two cases in the lumbosacral region. (**A**) Severe herpes zoster presentation in a patient who subsequently developed PHN, showing extensive hemorrhagic crusted lesions with confluent distribution. (**B**) Milder presentation in a patient with ACP only, displaying scattered erythematous lesions without hemorrhagic changes. ACP, acute phase pain; PHN, postherpetic neuralgia.

The mean age in the training cohort was 60 ± 12.9 years for the ACP group and 67 ± 9.6 years for the PHN group. In the test cohort, the mean age was 60 ± 13.5 years for the ACP group and 66 ± 9.0 years for the PHN group. Age differences between the ACP and PHN groups were significant in both cohorts (both *p* < 0.05). The study’s workflow is presented in Figure 1. A clinical presentation of herpes zoster cases in immunosuppressive patients is shown in Figure 2.

### 3.2. Univariate Analysis

In the training cohort, significant differences in the relationship between pain and rash (*p* < 0.001), cranial and cervical nerve involvement (*p* < 0.001), the prevalence of metabolic syndrome (*p* = 0.006) were found between the ACP and PHN groups. In the test cohort, no significant difference in the relationship between pain and rash (*p* = 0.206), cranial and cervical nerve involvement (*p* = 0.233) was observed. But the difference in the prevalence of metabolic syndrome was marginally significant (*p* = 0.057). No significant differences in the lesion distribution type were shown in both cohorts (both *p* > 0.05, Table 1).

**Figure 3:**
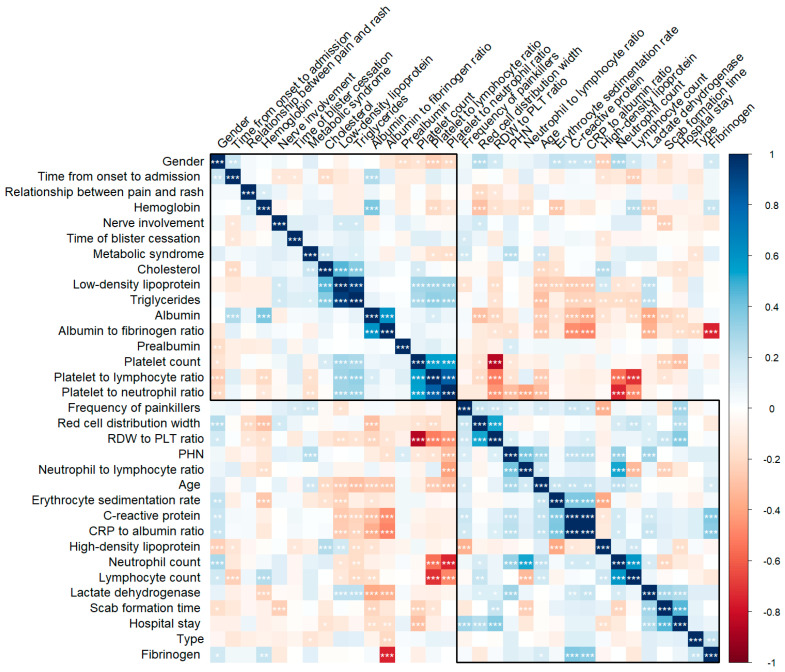
Correlation matrix of clinical and laboratory parameters in immunosuppressive patients with herpes zoster. Correlation matrix showing relationships between demographic, clinical, and laboratory parameters in immunosuppressive patients. Blue indicates positive correlation, red indicates negative correlation, and intensity reflects correlation strength. *, *p* < 0.05; **, *p* < 0.01; ***, *p* < 0.001; CRP, C-reactive protein; RDW, red cell distribution width.

In the training cohort, the PHN group had significantly higher LDH (*p* < 0.001), neutrophil count (*p* < 0.001), C-reactive protein (*p* = 0.004), prealbumin (*p* = 0.049), CAR (*p* = 0.004), and, while having lower platelet count (*p* = 0.045), AFR (*p* = 0.034), CAR (*p* = 0.034), PNR (*p* < 0.001) and PLR (*p* = 0.027). In the test cohort, only LDH (*p* = 0.012), neutrophil count (*p* = 0.007), PNR (*p* = 0.005) and NLR (*p* = 0.001) showed significant differences between the ACP and PHN groups. No significant differences were observed in other laboratory parameters such as hemoglobin, erythrocyte sedimentation rate, red cell distribution width, fibrinogen, lymphocyte count, high-density lipoprotein, low-density lipoprotein, triglycerides, cholesterol, RPR, CAR, or albumin between the groups in either cohort (all *p* > 0.05, Table 2). Correlation matrix of clinical and laboratory parameters in immunosuppressive patients with herpes zoster is shown in Figure 3.

### 3.3. Multivariate Analysis

Multivariate logistic regression identified several independent clinical predictors of PHN. The analysis revealed that metabolic syndrome (OR = 1.17, 95% CI: 1.02–1.34), age (OR = 1.12, 95% CI: 1.04–1.21), LDH (OR = 1.06, 95% CI: 1.02–1.10), and NLR (OR = 1.45, 95% CI: 1.18–1.78) were independently associated with higher odds of developing PHN. PLR and PNR were excluded from the multivariate model due to multicollinearity with NLR and limited incremental predictive value. The result of the multivariate logistic regression of the clinical predictors is shown in Table 3.

### 3.4. Nomogram Discrimination and Calibration

**Figure 4:**
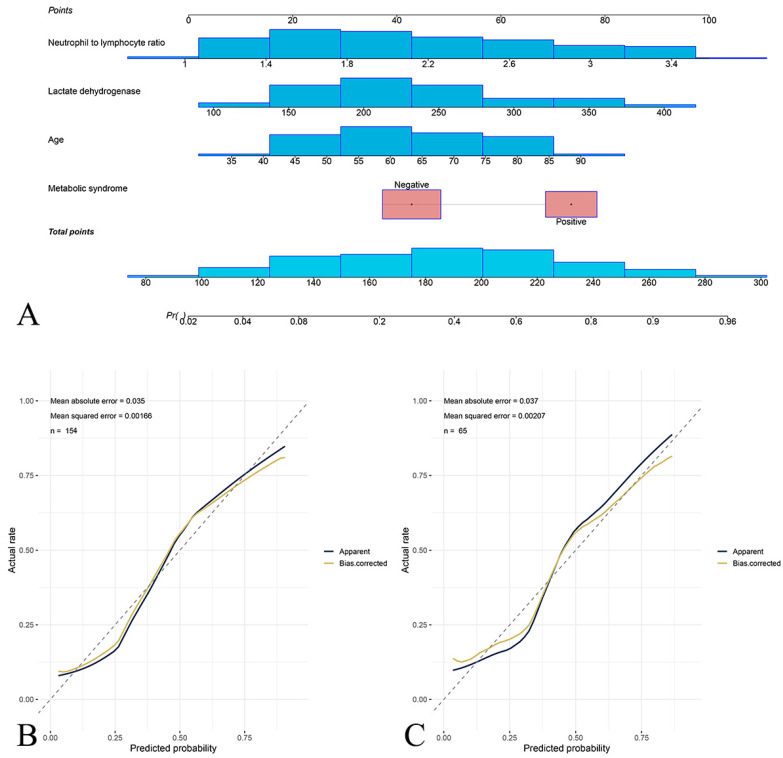
Nomogram for predicting postherpetic neuralgia risk and calibration curves for model validation. (**A**) Nomogram incorporating NLR, LDH, age, and metabolic syndrome status for individualized PHN risk prediction. For each patient, the value of each predictor is located on the corresponding axis; a vertical line is drawn to the ‘Points’ scale to obtain the individual score. The total points are summed and mapped to the bottom axis to estimate the predicted probability of PHN. Calibration curves for training (**B**) and test (**C**) cohorts demonstrating good agreement between predicted and observed PHN rates. The dashed line represents perfect (ideal) calibration, where predicted probability equals observed probability at every threshold. The proximity of the bias-corrected curve to the ideal dashed line in both cohorts confirms that the nomogram is well-calibrated. LDH, lactate dehydrogenase; NLR, neutrophil-to-lymphocyte ratio; PHN, postherpetic neuralgia.

**Figure 5:**
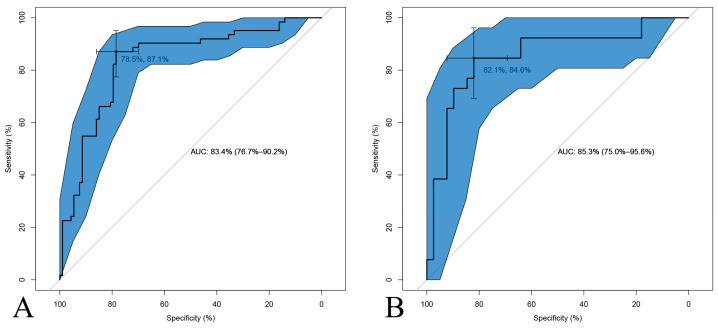
ROC curves of the nomogram for predicting PHN in immunosuppressive patients. (**A**) ROC curve in the training cohort and (**B**) ROC curve in the test cohort. The shaded blue region represents the 95% confidence interval of the ROC curve. The diagonal gray line indicates the reference line of no discrimination (AUC = 0.50). AUC, area under the receiver operating characteristic curve; PHN, postherpetic neuralgia.

A nomogram integrating the clinical predictors was constructed (Figure 4). For the training cohort, the nomogram achieved an AUC of 0.83 (95% CI 0.77–0.90) with a specificity of 0.78, sensitivity of 0.87, NPV of 0.90, and PPV of 0.73. For the test cohort, the nomogram achieved an AUC of 0.85 (95% CI 0.75–0.96) with a specificity of 0.82, sensitivity of 0.85, NPV of 0.89, and PPV of 0.76 (Figure 5). Calibration plots demonstrated good agreement between predicted and observed probabilities.

### 3.5. Clinical Usefulness

**Figure 6:**
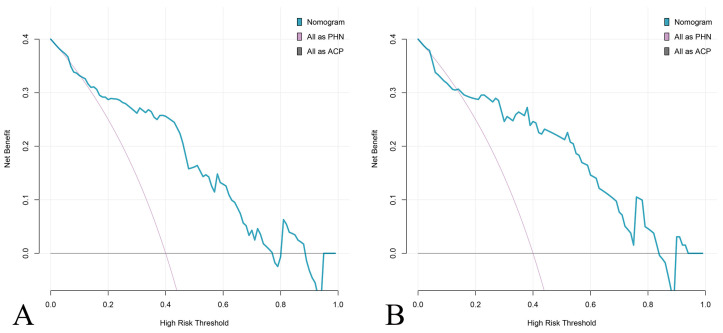
Clinical decision curve analysis for the PHN prediction nomogram. Decision curves showing net benefit of the nomogram-based prediction strategy compared to default strategies (treat all as PHN or treat none as PHN) in (**A**) training cohort and (**B**) test cohort. The nomogram (blue) provides superior net benefit across threshold probabilities of 0.1–0.9, confirming its clinical utility for risk-stratified patient management.

DCA results showed that the nomogram for predicting PHN added net benefit in both the training and test cohorts (Figure 6).

## 4. Discussion

This study provides an understanding of the risk factors contributing to PHN in patients under immunosuppressive conditions. The identification of age, metabolic syndrome, and LDH and NLR as significant predictors of PHN offer valuable insights for clinical practice. These findings underscore the importance of early recognition, comprehensive metabolic management, and targeted anti-inflammatory therapies in reducing the burden of PHN in patients under immunosuppressive conditions.

The occurrence of PHN with immunosuppressive conditions is related to the condition of the patient’s immune system. Previous studies showed that changes in the body’s immune system increase the risk of developing PHN [10]. Patients with immunosuppressive conditions exhibit a heightened susceptibility to herpes zoster reactivation [11]. This increased risk is primarily attributed to the compromised immune system’s reduced ability to control latent varicella-zoster virus reactivation. Once reactivated, varicella-zoster virus can cause more severe and prolonged neural damage in immunosuppressive individuals, leading to a higher incidence and severity of PHN [12].

In this study, age emerged as an influential factor for PHN occurrence. This observation suggests that older age contributes to the vulnerability to PHN. Some studies have indicated that PHN is more common in middle-aged and elderly individuals, particularly those over 50 years of age [13]. Advancing age is a possible reason for the decline in cellular and humoral immune functions. Furthermore, in elderly patients, the diminished ability to repair nerves ultimately increases the risk of developing PHN [13]. When herpes zoster occurs, the body is unable to effectively clear the varicella-zoster virus, resulting in persistently high levels of the virus and severe nerve damage. Additionally, the reduced capacity for nerve repair in elder adults further increases the risk of developing PHN.

Metabolic syndrome has been increasingly studied for its association with PHN. Recent research highlights both epidemiological links and underlying metabolic and immune mechanisms that may connect these conditions. Multiple studies indicate that type 2 diabetes mellitus (T2DM) is associated with a higher risk of developing PHN [14,15]. Impaired glucose tolerance is common in PHN patients, which suggests that PHN may serve as a marker for underlying metabolic disturbances. Research reveals that metabolic disturbances, especially in amino acid, lipid, and carbohydrate metabolism, are linked to PHN development [16]. Metabolomic studies also show that altered glutamate metabolism is found in PHN patients [17]. LDH is a key enzyme in glucose metabolism and is also widely recognized as a marker of tissue injury and inflammation. Elevated LDH levels are linked to altered metabolic state, poor prognosis and diminished immune response [18,19].

Inflammatory markers have been shown in numerous studies to more accurately reflect the body’s inflammatory and immune status compared to traditional single clinical indicators [20]. These composite markers have significant value in predicting the severity and prognosis of related diseases [21,22]. Additionally, among the inflammatory markers, NLR and PNR are all derived from key components of a complete blood count. They provide comprehensive information on immune pathways that reflect the body’s inflammatory status more accurately than single indicators. Generally, higher NLR levels are associated with poorer prognosis [23]. NLR has been reported to effectively predict patient prognosis and monitor disease progression in various conditions such as infectious diseases and sepsis. It has been reported that NLR is significantly higher in PHN patients [24]. In this study, the increase in NLR is positively correlated with the incidence of PHN, suggesting that the inflammation level is notably higher in the PHN group, which indicates that NLR has a predictive value for the occurrence of PHN.

Our nomogram findings can be compared with several recent PHN prediction models. Peng and Min developed a nomogram in 650 general herpes zoster patients and achieved AUC values of 0.94 and 0.90 in the training and validation sets [25]. Hu et al. built a prospective nomogram in 174 herpes zoster patients using age, female sex, prodromal pain, rash area, and acute-phase pain severity, with an AUC of 0.81 [26]. Both models relied on clinical and symptomatic variables. Liu and Chen constructed a nomogram for immunocompetent patients using age, CD4+/CD8+ ratio, Treg cell proportion, IL-6, TNF-α, and IL-10, achieving AUC values of 0.80 and 0.79 [22]. Liu et al. identified age, pain scores, CRP, and homocysteine as independent PHN predictors in 887 immunocompetent patients, with a combined AUC of 0.76 [27]. Compared with these studies, our nomogram achieves a comparable AUC (0.83 in training and 0.85 in test cohort) targeting immunosuppressed patients. Interestingly, age is a shared predictor across all models. However, rather than clinical symptoms or T cell subsets, our model uses routinely available laboratory markers such as NLR and LDH. This is consistent with the role of immune dysregulation demonstrated by Liu and Chen, but NLR is more accessible in daily practice than T cell subset quantification. These comparisons highlight the need for population-specific models, as the optimal predictive markers for PHN differ according to the immune background of the patient.

Our study has several limitations. First, the small sample size may limit the statistical power of the multivariate model and increase the risk of overfitting. Larger prospective cohorts would be needed to confirm the stability and the robustness of the nomogram. Second, differences in immunosuppressive regimens, comorbidity profiles, and clinical management practices across regions may affect the model’s transportability. Future international multicenter validation studies are needed to assess the external generalizability of our findings. Third, the degree and mechanism of immune compromise vary substantially across patients. This heterogeneity may influence the predictive weight of individual variables within the nomogram. Future studies stratified by immunosuppressive subtype would refine the predictive precision of the nomogram. Last, due to the retrospective design of the study, more severe cases may have been preferentially included in the cohort, which led to a higher incidence of PHN. This suggests that our nomogram might be better suited for predicting PHN risk in immunosuppressed populations rather than general populations.

## 5. Conclusions

PHN is prone to occur during the treatment of patients in an immunosuppressive state. The study highlights age, metabolic syndrome, LDH and NLR as key PHN predictors in immunosuppressive patients.

## Figures and Tables

**Table 1:** Clinical characteristics of the patients with immunosuppressive conditions after herpes zoster reactivation.

Term	Training Cohort			Test Cohort		
	ACP (*n* = 92)	PHN (*n* = 62)	*p*	ACP (*n* = 39)	PHN (*n* = 26)	*p*
Gender						
Female	57 (62.0%)	38 (61.3%)	1.000	31 (79.5%)	17 (65.4%)	0.327
Male	35 (38.0%)	24 (38.7%)		8 (20.5%)	9 (34.6%)	
Relationship between pain and rash						
Only rash	3 (3.3%)	14 (22.6%)	<0.001	1 (2.6%)	3 (11.5%)	0.206
Pain follows rash	27 (29.3%)	4 (6.5%)		11 (28.2%)	3 (11.5%)	
Pain precedes rash	7 (7.6%)	10 (16.1%)		6 (15.4%)	3 (11.5%)	
Simultaneously	55 (59.8%)	34 (54.8%)		21 (53.8%)	17 (65.4%)	
Nerve involvement						
Cranial and cervical	35 (38.0%)	8 (12.9%)	<0.001	11 (28.2%)	2 (7.7%)	0.233
Intercostal	10 (10.9%)	22 (35.5%)		9 (23.1%)	9 (34.6%)	
Limbs	7 (7.6%)	7 (11.3%)		4 (10.3%)	3 (11.5%)	
Lumbosacral	40 (43.5%)	25 (40.3%)		15 (38.5%)	12 (46.2%)	
Lesion distribution type						
Generalized	7 (7.6%)	1 (1.6%)	0.208	1 (2.6%)	0 (0%)	1.000
Non-generalized	85 (92.4%)	61 (98.4%)		38 (97.4%)	26 (100%)	
Metabolic syndrome						
Negative	61 (66.3%)	26 (41.9%)	0.006	27 (69.2%)	11 (42.3%)	0.057
Positive	31 (33.7%)	36 (58.1%)		12 (30.8%)	15 (57.7%)	
Age (y)	60 ± 12.9	67 ± 9.6	<0.001	60 ± 13.5	66 ± 9.0	0.034
Time of blister cessation (d)	7.1 ± 2.3	7.2 ± 2.4	0.109	7.1 ± 2.3	7.1 ± 2.2	1.000
Scab formation time (d)	6.0 ± 2.2	6.1 ± 1.2	0.679	6.5 ± 2.2	6.4 ± 1.3	0.71
Time from onset to admission (d)	4.6 ± 1.4	4.4 ± 2.0	0.559	4.7 ± 1.4	4.0 ± 1.1	0.052
Frequency of painkillers (Times)	11.9 ± 3.8	13.0 ± 4.3	0.104	11.0 ± 3.7	12.3 ± 3.7	0.167
Hospital stay (day)	7.4 ± 2.0	7.6 ± 1.5	0.616	7.4 ± 2.3	8.0 ± 1.6	0.311
Pain durations (week)	7.5 ± 2.6	19.4 ± 6.7	<0.001	7.1 ± 2.3	20.0 ± 6.7	<0.001

**Table 2:** Clinical laboratory tests of patients with immunosuppressive conditions after herpes zoster reactivation.

Term	Training Cohort			Test Cohort		
	ACP (*n* = 93)	PHN (*n* = 62)	*p*	ACP (*n* = 39)	PHN (*n* = 26)	*p*
Hemoglobin (g/L)	129 ± 18.5	125 ± 12.2	0.103	126 ± 16.7	122 ± 13.7	0.232
Platelet count (10^9^/L)	206 ± 51.6	189 ± 51.5	0.045	203 ± 60.8	191 ± 68.5	0.466
Lactate dehydrogenase (U/L)	214 ± 70.8	258 ± 53.0	<0.001	214 ± 62.6	248 ± 43.0	0.012
Erythrocyte sedimentation rate (mm/h)	21.2 ± 16.3	22.5 ± 14.8	0.596	20.5 ± 15.9	21.8 ± 14.0	0.713
Red cell distribution width (%)	47.8 ± 4.28	47.3 ± 7.23	0.641	47.7 ± 4.62	47.2 ± 7.52	0.765
Fibrinogen (g/L)	4.0 ± 0.65	4.1 ± 0.53	0.189	3.9 ± 0.63	4.1 ± 0.49	0.125
Albumin (g/L)	39.1 ± 4.8	37.6 ± 5.3	0.071	38.2 ± 5.0	38.2 ± 4.4	0.98
Prealbumin (mg/L)	221 ± 73.6	243 ± 61.5	0.049	250 ± 75.3	268 ± 65.9	0.323
Neutrophil count (10^9^/L)	3.8 ± 1.52	4.8 ± 1.36	<0.001	3.7 ± 1.48	4.7 ± 1.45	0.007
Lymphocyte count (10^9^/L)	2.0 ± 0.78	2.1 ± 0.66	0.85	2.0 ± 0.82	1.9 ± 0.51	0.717
C-reactive protein (mg/L)	11.1 ± 13.7	18.5 ± 16.8	0.004	13.1 ± 15.2	18.6 ± 12.9	0.125
High-density lipoprotein (mmol/L)	1.2 ± 0.24	1.2 ± 0.25	0.42	1.2 ± 0.20	1.2 ± 0.28	0.961
Low-density lipoprotein (mmol/L)	2.5 ± 0.65	2.6 ± 0.69	0.575	2.6 ± 0.72	2.5 ± 0.66	0.444
Triglycerides (mmol/L)	1.2 ± 0.68	1.3 ± 0.63	0.424	1.4 ± 0.77	1.2 ± 0.63	0.4
Cholesterol (mmol/L)	4.1 ± 0.89	4.2 ± 0.94	0.6	4.1 ± 0.89	4.1 ± 0.87	0.859
Neutrophil to lymphocyte ratio	1.9 ± 0.57	2.4 ± 0.66	<0.001	1.9 ± 0.61	2.5 ± 0.56	0.001
Platelet to lymphocyte ratio	117 ± 63.9	98.2 ± 40.0	0.027	121 ± 65.5	106 ± 44.9	0.263
Platelet to neutrophil ratio	65.3 ± 40.2	42.6 ± 19.0	<0.001	65.7 ± 40.4	43.8 ± 20.3	0.005
RDW to platelet ratio	0.2 ± 0.06	0.2 ± 0.10	0.080	0.2 ± 0.08	0.2 ± 0.10	0.415
CRP to albumin ratio	0.2 ± 0.37	0.5 ± 0.49	0.004	0.3 ± 0.40	0.5 ± 0.38	0.103
Albumin to fibrinogen ratio	9.9 ± 1.99	9.2 ± 2.01	0.034	10.0 ± 2.12	9.4 ± 1.77	0.228

**Table 3:** The result of the multivariate logistic regression of the clinical predictors.

Term	OR	*p*	CI
(Intercept)	0.25	0.008	0.08–0.68
Relationship between pain and rash	0.99	0.656	0.93–1.04
Nerve involvement	1.03	0.239	0.98–1.08
Metabolic syndrome	1.17	0.008	1.02–1.34
Age	1.12	0.040	1.04–1.21
Frequency of painkillers	1.00	0.648	0.98–1.02
Hemoglobin	1.00	0.733	0.99–1.00
Platelet	1.00	0.824	0.99–1.00
Lactate dehydrogenase	1.06	0.000	1.02–1.10
Prealbumin	1.00	0.059	1.00–1.00
Neutrophil count	0.97	0.445	0.88–1.06
C-reactive protein	1.01	0.687	0.97–1.04
Neutrophil to lymphocyte ratio	1.45	0.001	1.18–1.78
Platelet to lymphocyte ratio	1.00	0.156	0.99–1.00
Platelet to neutrophil ratio	1.00	0.482	0.99–1.01
CRP to albumin ratio	0.86	0.821	0.24–3.07
Albumin to fibrinogen ratio	1.01	0.622	0.97–1.04

## Data Availability

The original data presented in the study are openly available at https://github.com/jsyyky/PHN (accessed on 14 January 2026).

## Abbreviations

The following abbreviations are used in this manuscript:

ACP	acute phase pain
AUC	area under the receiver operating characteristic curve
CAR	c-reactive protein to albumin ratio
CI	confidence interval
CRP	c-reactive protein
DCA	decision curve analysis
LDH	lactate dehydrogenase
MLR	monocyte-to-lymphocyte ratio
NLR	neutrophil-to-lymphocyte ratio
NPV	negative predictive value
PHN	postherpetic neuralgia
PLR	platelet-to-lymphocyte ratio
PPV	positive predictive value
PNR	platelet-to-neutrophil ratio
RDW/Hb	red cell distribution width to hemoglobin ratio
ROC	receiver operating characteristic
VAS	visual analogue scale
VIF	variance inflation factor
